# Sleep pattern gender differences and fragmentation in postpartum parents of twins

**DOI:** 10.5935/1984-0063.20200101

**Published:** 2021

**Authors:** Elizabeth G Damato, Christopher J Burant, Jennifer A Brubaker, Michael J Decker

**Affiliations:** 1 Case Western Reserve University, Frances Payne Bolton School of Nursing - Cleveland - Ohio -United States.; 2 Case Western Reserve University, Department of Physiology & Biophysics - Cleveland - Ohio - United States.; 3 Case Western Reserve University, Department of Pulmonary, Critical Care, Sleep Medicine - Cleveland - Ohio -United States.; 4 Louis Stokes Cleveland VA Medical Center, Geriatric Research, Education, and Clinical Center - Cleveland -Ohio - United States.; 5 Cleveland Clinic Foundation, General Pediatrics, Lorain Family Health Center - Cleveland - Ohio -United States.

**Keywords:** Sleep Deprivation, Multiple Birth Offspring, Maternal and Child Health, Paternal Behavior

## Abstract

**Objective:**

Parents of newborn twins are at risk for both shortened sleep duration and sleep discontinuity. The purpose of this study was to characterize weekday and weekend sleep duration, sleep continuity, and awakenings in both mothers and fathers of newborn twins during the first 3 months at home.

**Material and Methods:**

Sleep-wake parameters were assessed at two time points using self-report diaries and actigraphy in 75 families with newborn twins. To assess sleep on weekdays and weekends with minimal subject burden, actigraphy recordings of both parents commenced at 9:00 p.m. Saturday and terminated at 9:00 p.m. Tuesday.

**Results:**

Mean sleep duration over 24 hours for parents of twins ranged between 6.7 and 7.5 hours during the first 3 months postpartum and did not significantly differ on weekdays or weekends for mothers. Weekend sleep was more fragmented for fathers at both one month and three months with more awakenings, compared to weekday sleep. Mothers had more fragmented night sleep compared to fathers at one month. In contrast, at three months postpartum fathers had shorter total sleep time and night sleep time, but fewer night awakenings on weekdays than mothers. No differences were observed in weekend sleep duration or sleep patterns between mothers and fathers at three months.

**Discussion:**

Consolidated sleep periods for both parents averages three hours or less during the first three months postpartum and sleep for both parents is fragmented. In families with newborn twins, the extent of sleep disruption for mothers and fathers is similar.

## INTRODUCTION

Postpartum families are at risk for disruptions in sleep patterns, including fragmented and/or shortened sleep. Chronic poor quality sleep contributes to declines in cognitive function^1^, excessive daytime sleepiness^2^, and fatigue, all of which can adversely affect parents' ability to meet childrearing demands^3,4^. Poor sleep is linked to depressed mood in new mothers^4,5^. Depressed mothers are less likely to follow safe infant sleep practices, less likely to always use a car seat, more likely to feed their infants juice or cereal before 4 months of age, and more likely to utilize emergency rooms for health care visits^6^. Maternal postpartum depression is also linked to less affectionate and lower emotional attunement with the infant^7^.

Meeting the 24-hour feeding and caretaking needs of a newborn infant leads to inescapable factors contributing to sleep disruption for new parents. These demands are further escalated with twins, particularly given the increased risk for premature birth. Over 79% of live-born twins in the U.S. are delivered preterm at less than 37 weeks gestation, and 65% are classified as low birth weight (<2,500 grams)^8^. Preterm infants are more fragile, require more vigilant care^9^, and show preference for nighttime wakefulness^10^, further placing parents of twins at risk for disruptions in sleep. In contrast to families with singletons, parents of multiples spend the same amount of time caring for each infant, thereby doubling their time spent caring for twins^11^. Free time that a mother may have to rest is necessarily allocated to meet the needs of the second infant. Additionally, many mothers are recovering from the sequelae of antepartum bed rest used to treat complications of their high-risk twin pregnancies, including muscle atrophy and cardiovascular deconditioning^12^. As many as 66% are recovering from cesarean deliveries^13^.

While mothers are typically the primary care provider for a newborn infant, fathers are described as their major source of support. Many of today's fathers actively participate in infant care, yet even fathers of singleton infants report disrupted sleep when the infant or their partner is awake during the night. Despite the availability of paternal leave, most families cannot afford multiple weeks of unpaid leave^14^, particularly when trying to meet the additional financial demands of two new family members. Often the only employed parent, fathers need to return to employment responsibilities soon after the birth of twins, precluding most opportunities to recapture lost sleep during the day. Furthermore, as newborn care for two infants must occur each night and each day, weekends may not offer respite and opportunities for "catch-up" sleep.

While common sense indicates that parents of twins are likely to perceive their sleep as inadequate, few studies have characterized the impact of caring for twin infants upon both maternal and paternal sleep patterns. One previous report in a small sample of 8 families found fathers of newborn twins experienced significantly less night sleep than mothers (5.4 versus 6.2 hours) at 2 weeks post discharge^15^. Thus, the aim of this study was to extend those findings by characterizing sleep duration, sleep continuity, and awakenings in a larger sample of mothers and fathers of newborn twin infants during the workweek and on weekends.

## MATERIAL AND METHODS

Research subjects were participants in a larger prospective descriptive comparative design study of sleep and depression in families of newly delivered twins. Study procedures were approved by the appropriate ethics committee at each recruitment hospital (Fairview Hospital, IRB# 05-11-029b; Hillcrest Hospital, IRB# 05-11-029-12; MetroHealth Medical Center IRB# 08-00506; University Hospitals Cleveland Medical Center, IRB# 03-07-29) and were performed in accordance with the ethical standards laid down in the 1964 Declaration of Helsinki and its later amendments. Both mothers and fathers separately provided written informed consent.

The study was advertised at prenatal childbirth classes held for expectant parents of multiples. Recruitment and enrollment occurred in the postpartum units of four hospitals in the Midwestern United States over a two-year period. Eligibility criteria were: mothers who were newly delivered primiparous and multiparous postpartum women, able to read and write English, and were at least 18 years of age with telephone access. Eligible women could have no more than two other children at home and needed to report that any older child regularly slept through the night. Fathers were required to reside in the same household as the mother and twins. To avoid confounding of lengthy neonatal hospital stays, twins were required to be ≥33 weeks gestational age at birth. Exclusion criteria included: night shift employment for either parent, diagnosed sleep disorder, illicit drug use, alcohol abuse, or clinically diagnosed depression. Families also were excluded if either twin had congenital anomalies, or required home cardiorespiratory monitoring or gavage feedings after hospital discharge.

### Measures

Self-report and actigraphy-derived correlates of sleep-wake were obtained from parents at approximately 2 weeks and 12 weeks following the hospital discharge of the twins. Mothers and fathers wore the Octagonal Basic Motionlogger wrist actigraph (Ambulatory Monitoring Inc., Ardsley, NY) on the non-dominant wrist, with activity counts calculated for 1-minute epochs stored in the time above threshold (TAT) data mode. An investigator-developed sleep diary was used to assist in the interpretation of objectively recorded actigraphy data. To decrease burden, subjects wore actigraphs and completed sleep diaries for three consecutive days with evening starting times (Saturday 9:00 p.m.) and ending times (Tuesday 9:00 p.m.). An evening start time simplified the protocol for busy parents of twins, while reducing the number of sleep diaries requiring completion during each data collection period. Furthermore, this protocol provided representative data of sleep patterns by including one weekend day and two weekdays. Along with documenting sleep patterns in their diaries, parents were instructed to press the wrist actigraph event marker at the beginning and end of each night sleep period. Actigraphy data were analyzed using ActionW^©^ manufacturer-compatible software (version 2.6).

Total sleep time was calculated from actigraphy data as the total sleep minutes in each 24-hour period. Night sleep was defined as sleep occurring between the times parents pressed the actigraph event marker to signify the beginning and end of each night's sleep period. Actigraphic data were used to identify the number of awake episodes equal to or greater than 5 minutes duration and the longest uninterrupted sleep episode during the night sleep period.

Investigator-developed parental information forms were used to collect demographic data such as age, years of education, race and ethnicity, employment hours, family income, delivery method, infant birth weight and gestational age, and number of other children in the home.

### Statistical analysis

Ninety two-parent families of twins were studied. Families for whom complete actigraphy data were available from the mother at one month postpartum (n=75) are included in this report. Descriptive statistics were calculated for demographic variables. Analyses were exploratory in nature and included paired t-tests for comparison of weekday and weekend sleep for each parent and for comparisons between mothers and fathers. Comparisons were made at one month and three months postpartum. The significance level was set at *p*<5% (two-sided), without correction for multiple comparisons in order to assess individual sleep pattern variables and to avoid missing possible differences between parents that may be worthy of further study^16^. Data management and statistical analyses were performed with SPSS version 26 (IBM SPSS Inc., Armonk, NY). Effect sizes were calculated using G*Power 3.1^17^.

## RESULTS

### Sample

The majority of participants were White/non-Hispanic, over age 30, and well-educated. The twins were the only children for 44% of the families, and over 70% of the mothers were recovering from a cesarean delivery. Detailed sample demographics are presented in Table 1 for the full data set of 75 mother-father pairs.

**Table 1.** Sample Demographics.

	Sample Demographics		
	Mothers (n=75)		Fathers (n=75)*
**Age in years (M ± SD)**	32.8 ± 5.2		33.9 ± 5.4
**Education in years (M ± SD)**	15.9 ± 2.4		16.2 ± 2.8
**Race**	82.7% White 9.3% Black/African American 5.3% Asian 1.3% Mixed (n=1) 1.3% Missing (n=1)		78.7% White 14.7% Black/African American 5.3% Asian 1.3% Missing (n=1)
**Ethnicity**	96.0% non-Hispanic		94.7 % non-Hispanic
**Employment**	1.3% full/part time at 1 month 42.7% full/part time at 3 months		86.7% full/part time at 1 month 86.7% full/part time at 3 months
**Other Children**	44.0% No other children 42.7% One other child 13.3% Two other children		
**Method of Birth**	29.3% Vaginal birth 70.7% Cesarean birth		
		**Infant Demographics**		
**Birth Weight in grams (M ± SD)** ** Twin A** ** Twin B**		2605.7 ± 376.8 2541.2 ± 397.6		
**Gestation at birth in weeks (M ± SD)**		36.5 ± 1.4		
**Age in days (M ± SD)** ** Two weeks post hospital discharge of twins** ** Twelve weeks post hospital discharge of twins**		28.9 ± 10.2 91.7 ± 7.8		

*M=Mean; SD=Standard deviation.*

* *Actigraphy data were available for only 68 of the 75 fathers who participated*

### Sleep at one month postpartum

Actigraphy data were available at one month postpartum for 75 mothers and 68 fathers. Weekday and weekend sleep pattern variables were examined separately for mothers and for fathers. Mean total sleep time over 24 hours for weekdays and weekends was 419.8 minutes (~7.0 hours) for mothers and 414.5 minutes (6.9 hours) for fathers at one month postpartum. Night sleep time and number of night sleep awakenings for mothers did not differ between weekdays and weekends, yet mothers had longer average night sleep episodes on weekends compared to weekdays (132.8 minutes/2.21 hours vs. 119.4 minutes/1.99 hours, respectively). Fathers had longer night sleep time on weekends (381.1 minutes/6.35 hours) compared to weekdays (358.7 minutes/5.98 hours), but with more frequent night awakenings. Table 2 details weekday and weekend comparisons separately for each parent. Effect sizes are also provided in Table 2. For the sample of 75 mothers, an alpha of 0.05, an effect size of 0.28 was needed to reach a power of 0.80. For the sample of 68 fathers, an alpha of 0.05, an effect size of 0.30 was needed to reach a power of 0.80.

**Table 2.** Weekday-Weekend Comparisons at One Month for Each Parent.

Weekday-Weekend Comparisons at One Month								
	Mothers				Fathers			
	Mean Minutes (± SD) n=75				Mean Minutes (± SD) n=68			
	**Weekday**	**Weekend**	*t(df) p*	*Effect size †*	**Weekday**	**Weekend**	*t(df) p*	*Effect size †*
Total Sleep Time/24 hours (minutes)	413.8 ± 67.0	425.7 ± 80.1	-1.61 (74) p = 0.112	0.16	404.2 ± 87.4	424.8 ±106.7	-1.64 (67) p = 0.106	.021
Night Sleep Time (minutes)	354.3 ± 72.5	362.5 ± 93.1	-1.03 (74) p = 0.309	0.10	358.7 ± 77.0	381.1 ± 93.7	-2.08 (67)* **p = 0.042**	0.26
Longest Night Sleep Episode (minutes)	119.4 ± 31.8	132.8 ± 59.4	-2.01 (74)* **p = 0.049**	0.26	137.2 ± 58.8	140.1 ± 79.6	-0.30 (67) p = 0.762	0.04
Number of Night Awakenings	5.61 ± 2.4	5.05 ± 2.9	1.82 (74) p = 0.073	0.21	3.90 ± 2.6	4.81 ± 3.1	-2.25 (67)* **p = 0.027**	0.32

* *p < 0.05; M=Mean; SD=Standard deviation; t=t statistic; df = degrees of freedom*

† Effect sizes > 0.30 have a power > 0.80.

Actigraphy data were available for 68 mother-father dyads at one month postpartum. Total sleep time and night sleep time did not differ between parents at one month. Compared to mothers, the average duration of the longest night sleep episode was longer for fathers on weekdays and fathers had fewer night awakenings. Weekend sleep did not differ between parents (Table 3). Effect sizes are also provided in Table 3. For the sample of 68 mother-father dyads, an alpha of 0.05, an effect size of 0.30 was needed to reach a power of 0.80.

**Table 3.** Mother-Father Comparisons of Sleep Variables on Weekdays and Weekends at One Month.

Comparisons of 68 Mother-Father Dyads at One Month								
	Weekday Mean Minutes (± SD)				Weekend Mean Minutes (± SD)			
	**Mothers**	**Fathers**	*t(df) p*	*Effect size †*	**Mothers**	**Fathers**	*t(df) p*	*Effect size †*
Total Sleep Time/24 hours (minutes)	417.1 ± 68.9	404.2 ± 87.4	0.99 (67) p = 0.328	0.16	432.5 ± 79.7	424.8 ± 106.7	0.51 (67) p = 0.611	0.08
Night Sleep Time (minutes)	356.7 ± 74.2	358.7 ± 77.0	-0.18 (67) p = 0.857	0.03	368.9 ± 93.9	381.1 ± 93.7	-0.89 (67) p = 0.378	0.13
Longest Night Sleep Episode (minutes)	118.8 ± 31.6	143.8 ± 67.8	-2.78 (67)* **p = .007**	0.43	135.2 ± 60.7	140.1 ± 80.0	-0.44 (67) p = 0.660	0.07
Number of Night Awakenings	5.65 ± 2.4	3.90 ± 2.6	4.63 (67)* **p < 0.001**	0.70	5.18 ± 3.0	4.81 ± 3.1	0.75 (67) p = 0.456	0.12

* *p < 0.05; M=Mean; SD=Standard deviation; t=t statistic; df = degrees of freedom*

† Effect sizes > 0.30 have a power > 0.80.

### Sleep at three months postpartum

Actigraphy data were available at three months postpartum for 66 mothers and 60 fathers. Mean total sleep time for weekdays and weekends was 437.8 minutes (7.3 hours) for mothers and 433.5 minutes (7.2 hours) for fathers at three months postpartum. Total sleep time, night sleep time, the average duration of the longest night sleep episode, and the number of awakenings did not differ on weekdays compared to weekends for mothers. Fathers had significantly longer total sleep time (~31 minutes) and night sleep time (~29 minutes) on weekends compared to weekdays, despite more frequent weekend night awakenings (Table 4). Effect sizes are also provided in Table 4. For the sample of 60 fathers, an alpha of 0.05, an effect size of 0.32 was needed to reach a power of 0.80. There were no significant findings for mothers; effect sizes were small and did not reach a power of 0.80.

**Table 4.** Weekday-Weekend Comparisons at Three Months for Each Parent.

Weekday-Weekend Comparisons at Three Months								
	Mothers Mean Minutes (±SD) n=66				Fathers Mean Minutes (±SD) n=60			
	**Weekday**	**Weekend**	*t(df) p*	*Effect size †*	**Weekday**	**Weekend**	*t(df) p*	*Effect size †*
Total Sleep Time/24 hours (minutes)	438.7 ± 76.0	436.9 ± 89.4	-0.15(65) p = 0.878	0.02	418.2 ± 80.6	448.8 ± 95.8	-2.68 (59)* **p = 0.009**	0.79
Night Sleep Time (minutes)	397.9 ± 65.7	395.0 ± 80.8	0.26 (65) p = 0.797	0.04	381.0 ± 72.0	409.5 ± 81.1	-2.75 (59)* **p = 0.008**	0.37
Longest Night Sleep Episode (minutes)	177.2 ± 78.2	158.2 ± 67.0	1.66 (65) p = 0.102	0.26	164.2 ± 70.0	153.6 ± 78.1	0.96 (59) p = 0.343	0.14
Number of Night Awakenings	3.98 ± 2.3	3.88 ± 2.6	0.32 (65) p = 0.754	0.04	3.22 ± 2.0	4.31 ± 3.2	-2.64 (59)* **p = 0.011**	0.39

* *p < 0.05; M=Mean; SD=Standard deviation; t=t statistic; df = degrees of freedom*

† Effect sizes > 0.30 have a power > 0.80.

Sleep pattern variables were compared at three months for the 59 mother-father dyads with actigraphy data. During weekdays at three months, fathers had less total sleep, shorter night sleep, and fewer night awakenings than mothers. No differences were found between mothers and fathers on any of the measured sleep pattern variables on weekends (Table 5). Effect sizes are also provided in Table 5. For the sample of 59 mother-father dyads, an alpha of 0.05, an effect size of 0.33 was needed to reach a power of 0.80.

**Table 5.** Mother-Father Comparisons of Sleep Variables on Weekdays and Weekends at Three Months.

Comparisons of 59 Mother-Father Dyads at Three Months								
	Weekday Mean Minutes (± SD)				Weekend Mean Minutes (± SD)			
	**Mothers**	**Fathers**	*t(df) p*	*Effect size †*	**Mothers**	**Fathers**	*t(df) p*	*Effect size †*
Total Sleep Time/24 hours (minutes)	443.4 ± 75.7	417.3 ± 80.7	2.14 (58)* *p* = 0.037	0.33	443.2 ± 85.7	441.7 ± 102.1	-0.11 (58) *p *= 0.912	0.02
Night Sleep Time (minutes)	401.3 ± 64.3	379.6 ± 71.9	2.18 (58)* *p* = 0.033	0.32	400.5 ± 77.2	407.8 ± 80.7	-0.69 (58) *p* = 0.491	0.09
Longest Night Sleep Episode (minutes)	182.7 ± 80.4	161.1 ± 66.3	1.86 (58) *p* = 0.069	0.29	162.1 ± 67.1	149.8 ± 72.8	1.05 (58) *p* = 0.299	0.18
Number of Night Awakenings	3.98 ± 2.3	3.23 ± 2.0	2.39 (58)* *p* = 0.020	0.35	3.73 ± 2.5	4.33 ± 3.2	-1.47 (58) *p* = 0.148	0.21

* **p < 0.05;** *M=Mean; SD=Standard deviation; t=t statistic; df = degrees of freedom*

† Effect sizes > 0.30 have a power > 0.80.

## DISCUSSION

Past studies have described sleep in postpartum mothers, but most samples have excluded mothers who deliver more than one infant. Very few investigations of postpartum sleep have included fathers. This is the first study to quantify total sleep time, night sleep duration, and the extent of sleep fragmentation on mothers and fathers of twins, a subset of postpartum families at high risk for disrupted and shortened sleep.

We found that sleep differed on weekdays and weekends for parents of twins, but differently for each parent. At one month postpartum, mothers had more awakenings and shorter bouts of consolidated night sleep on weekdays, suggesting that mothers may have protected the sleep of an employed father (the effect sizes were moderate to large). Weekend sleep was better than weekday sleep (mothers had longer consolidated sleep and fathers had longer night sleep duration despite more frequent awakenings), but no significant differences were found between parents.

At three months postpartum, weekend sleep had improved for fathers, with more total sleep time and more night sleep time compared to weekdays. The significant difference between weekday and weekend 24-hour total sleep for fathers was supported by a large effect size. Yet fathers had more frequent night awakenings on weekends, suggesting that they may have been contributing more to the nighttime care of the infants in order to provide respite for mothers on days when they may not have needed to leave for paid employment. Sleep continuity remained compromised for both parents at three months postpartum, with the longest night sleep episode hovering at or under three hours. Weekday total sleep and night sleep duration was shorter for fathers compared to mothers, although mothers had more frequent night awakenings. No differences were found on any of the sleep measures between parents on weekends at three months; night sleep for both parents remained fragmented with multiple awakenings.

A wide range of variability exists in previously reported sleep duration for mothers of healthy term singleton infants. Total 24 hour sleep times for mothers of twins in this study, approximately 420 minutes at one month and ~438 minutes at three months, were similar to the total 24 hours sleep time of 424.8 minutes reported for mothers of singleton infants^18^ assessed with actigraphy at approximately 7 weeks postpartum. Conversely, mothers of twins in our sample experienced much shorter total sleep over 24 hours than the 9.7 hours (~582 minutes) reported in an actigraphic study of mothers of healthy term infants studied at a mean of 17 days postpartum^19^.

Our study found night sleep to be shorter and more fragmented for mothers of twins compared to most previous reports in the literature for mothers of singletons. Using detailed sleep diaries, mothers of singleton infants reported longer night sleep times, averaging 445.8 minutes at 1.5 months postpartum and 439.6 minutes at 3 months postpartum^2^. Other studies using actigraphy reported similar maternal night sleep durations of 412.8 minutes around the 7^th^ postpartum week^18^ and 408 to 436.1 minutes around the 11^th^ postpartum week^20,21^. In contrast, mothers of twins in our study reported night sleep times averaging 358 minutes at one month, and approximately 396 minutes at three months, similar to night sleep times reported by Volkovich et al. (2018)^22^ and Tikotzky et al. (2015)^23^ in actigraphic studies of mothers of singletons at 3 months postpartum. We found the average duration of the longest night sleep episode in mothers of twins to be approximately an hour shorter, with a higher number of night awakenings than previously reported for mothers of singletons^2,23^.

A prior actigraphic study of postpartum sleep in mother-father dyads of singleton infants reported that fathers had significantly less sleep than mothers when studied at approximately 7 weeks postpartum^18^. In contrast, our study showed no differences in total 24 hours sleep duration or night sleep times between mothers and fathers at one month postpartum, although fathers of twins in our sample had significantly less sleep than mothers at three months postpartum. Unexpectedly, fathers in our sample experienced longer night sleep than two previous reports of sleep in fathers of singleton infants^3,18^. Possible explanations may include differences in assessment methods (survey vs. actigraphy) or differences in the percentage of fathers who were employed. Fathers of twins in our sample experienced more frequent night awakenings than reported in the survey of Australian fathers^3^.

Sufficient sleep duration and sleep continuity are considered two key dimensions of healthy sleep^24^. Although the optimal duration of total sleep over 24 hours is in debate, recommended sleep duration for most adults is between 7 to 9 hours^25,26^. Good quality sleep is also influenced by timing, with sleep during the nighttime hours in association with circadian rhythms being most beneficial^27^. Sleep in parents of newborn twins is not only shortened, but fraught with multiple awakenings. This is important because night awakenings are particularly detrimental to sleep quality^5,28^. Findings from the present study highlight the extent of night sleep fragmentation for parents of twins.

### Implications

This study's findings provide evidence that paternal sleep is similarly or even more disrupted than maternal sleep in families caring for newborn twins. Economic necessity forces many new parents back into the workforce soon after the birth of twins and it is important for employers to recognize the increased risks to health and safety of sleep restricted workers^3^. Although new parents are counseled to obtain adequate sleep, few empirically supported strategies exist that effectively decrease sleep restriction for new parents. Moreover, no tailored strategies exist to meet the unique needs of parents raising twins. Findings from this study contribute to an understanding of the extent of sleep disturbances for parents of twins and can be used to develop future interventional studies that minimize sleep disruption.

### Strengths and limitations

This study is the first to provide empirical evidence of the degree of sleep disruption in both mothers and fathers of twins. Strengths of this study included the large sample size, the measurement of paternal as well as maternal sleep, the longitudinal study design, and the use of actigraphy for objective measurement of sleep. The study was limited by the short actigraph recording period, a concession we felt necessary to implement in order to avoid imposing additional burdens on parents caring for two newborn infants. To decrease inadvertent bias during this shorter measurement period, we avoided data collection on holidays or weekends where parents were traveling or had houseguests that may have altered their typical sleep patterns.

Study attrition was relatively low, although sample size did decrease over time due to participant desire to withdraw from the study, technical failure of the actigraph devices, and non-compliance with the study protocol for the full 3-day recording period. Additionally, we did not account for the influence of confounders of parent sleep, such as whether the infants were breast-fed or formula fed. Infant feeding was complex for families of twins in this study; in many families twins were not fed by the same method nor the same number of times. Finally, the sample was somewhat limited by a lack of racial, ethnic, and economic diversity, such that results may not be generalizable to all families of twins.

## CONCLUSION

Sleep restriction is experienced by both parents regardless of day of week. The short duration of uninterrupted night sleep episodes and frequency of night awakenings highlight the fragmented nature of sleep for postpartum parents of twins.

## References

[1] Banks S, Dorrian J, Basner M, Dinges DF, Kryger M, Roth T, Dement WC (2017). Principles and practice of sleep medicine.

[2] Filtness AJ, MacKenzie J, Armstrong K (2014). Longitudinal change in sleep and daytime sleepiness in postpartum women. PLoS One.

[3] Mellor G, St John W (2012). Fatigue and work safety behavior in men during early fatherhood. Am J Mens Health.

[4] Stremler R, Sharkey KM, Wolfson AR, Kryger M, Roth T, Dement WC (2017). Principles and practice of sleep medicine.

[5] Bei B, Coo S, Trinder J (2015). Sleep and mood during pregnancy and the postpartum period. Sleep Med Clin.

[6] Balbierz A, Bodnar-Deren S, Wang JJ, Howell EA (2015). Maternal depressive symptoms and parenting practices 3-months postpartum. Matern Child Health J.

[7] Slomian J, Honvo G, Emonts P, Reginster JY, Bruyere O (2019). Consequences of maternal postpartum depression: a systematic review of maternal and infant outcomes. Womens Health.

[8] Martin JA, Hamilton BE, Osterman MJK, Driscoll AK (2019). Births: final data for 2018.

[9] Phillips-Pula L, Pickler R, McGrath JM, Brown LF, Dusing SC (2013). Caring for a preterm infant at home: a mother's perspective. J Perinat Neonatal Nurs.

[10] Giganti F, Fagioli I, Ficca G, Cioni G, Salzarulo P (2001). Preterm infants prefer to be awake at night. Neurosci Lett.

[11] Holditch-Davis D, Roberts D, Sandelowski M (1999). Early parental interactions with and perceptions of multiple birth infants. J Adv Nurs.

[12] Maloni JA, Margevicius SP, Damato EG (2006). Multiple gestation: side effects of antepartum bed rest. Biol Res Nurs.

[13] Sparks TN, Caughey AB, Cheng YW (2017). Cesarean delivery for twins: how have national rates changed over time?. Am J Obstet Gynecol.

[14] Insana SP, Garfield CF, Montgomery-Downs HE (2014). A mixed-method examination of maternal and paternal nocturnal caregiving. J Pediatr Health Care.

[15] Damato EG, Burant C (2008). Sleep patterns and fatigue in parents of twins. J Obstet Gynecol Neonatal Nurs.

[16] Armstrong RA (2014). When to use the Bonferroni correction. Ophthalmic Physiol Opt.

[17] Faul F, Erdfelder E, Lang AG, Buchner A (2007). G*Power 3: a flexible statistical power analysis program for the social behavioral and biomedical sciences. Behav Res Methods.

[18] Insana SP, Montgomery-Downs HE (2013). Sleep and sleepiness among first-time postpartum parents: a field- and laboratory-based multimethod assessment. Dev Psychobiol.

[19] Tsai SY, Thomas KA (2012). Sleep disturbances and depressive symptoms in healthy postpartum women: a pilot study. Res Nurs Health.

[20] Goyal D, Gay C, Lee K (2009). Fragmented maternal sleep is more strongly correlated with depressive symptoms than infant temperament at three months postpartum. Arch Womens Ment Health.

[21] Insana SP, Montgomery-Downs HE (2010). Maternal postpartum sleepiness and fatigue: associations with objectively measured sleep variables. J Psychosom Res.

[22] Volkovich E, Bar-Kalifa E, Meiri G, Tikotzky L (2018). Mother-infant sleep patterns and parental functioning of room-sharing and solitary-sleeping families: a longitudinal study from 3 to 18 months. Sleep.

[23] Tikotzky L, Sadeh A, Volkovich E, Manber R, Meiri G, Shahar G (2015). Infant sleep development from 3 to 6 months postpartum: links with maternal sleep and paternal involvement. Monogr Soc Res Child Dev.

[24] Buysse DJ (2014). Sleep health: can we define it? Does it matter?. Sleep.

[25] Chaput JP, Dutil C, Sampasa-Kanyinga H (2018). Sleeping hours: what is the ideal number and how does age impact this?. Nat Sci Sleep.

[26] Shen X, Wu Y, Zhang D (2016). Nighttime sleep duration, 24-hour sleep duration and risk of all-cause mortality among adults: a meta-analysis of prospective cohort studies. Sci Rep.

[27] Carskadon MA, Dement WC, Kryger M, Roth T, Dement WC (2017). Principles and practice of sleep medicine.

[28] Gress JL, Chambers AS, Ong JC, Tikotzky L, Okada RL, Manber R (2010). Maternal subjective sleep quality and nighttime infant care. J Reprod Infant Psychol.

